# The permeability of fractured rocks in pressurised volcanic and geothermal systems

**DOI:** 10.1038/s41598-017-05460-4

**Published:** 2017-07-21

**Authors:** A. Lamur, J. E. Kendrick, G. H. Eggertsson, R. J. Wall, J. D. Ashworth, Y. Lavallée

**Affiliations:** Department of Earth, Ocean and Ecological Sciences, University of Liverpool, 4 Brownlow Street, L69 3GP Liverpool, United Kingdom

## Abstract

The connectivity of rocks’ porous structure and the presence of fractures influence the transfer of fluids in the Earth’s crust. Here, we employed laboratory experiments to measure the influence of macro-fractures and effective pressure on the permeability of volcanic rocks with a wide range of initial porosities (1–41 vol. %) comprised of both vesicles and micro-cracks. We used a hand-held permeameter and hydrostatic cell to measure the permeability of intact rock cores at effective pressures up to 30 MPa; we then induced a macro-fracture to each sample using Brazilian tensile tests and measured the permeability of these macro-fractured rocks again. We show that intact rock permeability increases non-linearly with increasing porosity and decreases with increasing effective pressure due to compactional closure of micro-fractures. Imparting a macro-fracture both increases the permeability of rocks and their sensitivity to effective pressure. The magnitude of permeability increase induced by the macro-fracture is more significant for dense rocks. We finally provide a general equation to estimate the permeability of intact and fractured rocks, forming a basis to constrain fluid flow in volcanic and geothermal systems.

## Introduction

The storage and transport of fluids in the Earth’s crust is of primary importance for our understanding of georesources and geohazards. In volcanic settings, fluids both circulate in hydrothermal reservoirs^1^ commonly exploited for geothermal energy, and drive magma ascent and volcanic eruptions^2–4^. Better constraints of how fluids are transported in these systems will help define more accurate models, which in turn could lead to enhanced geothermal exploitation as well as improved prediction of volcanic eruptions.

All materials are inherently permeable, as permeability expresses either the diffusion speed at a molecular level or the capacity of a porous structure, at macroscopic level, to carry fluid flow. The permeability of rocks has been central to an extensive body of geoscientific studies since the early efforts of Darcy^5, 6^ and is often described in terms of its relationship to porosity^7–10^. In pursuit of a simple model constraining laminar flow in conduits, the Kozeny-Carman^11–14^ relationship, or modifications therof, can commonly be employed to explain that permeability increases non-linearly as a function of porosity for a wide range of rocks^15–22^. This equation describes the evolution of the permeability-porosity relationship by applying a coefficient dependent on the dominant conduit geometry controlling the fluid flow, namely tubular (connected pores) or planar (cracks) conduits^23, 24^. Previous experimental studies have invoked the existence of a percolation threshold for explosive volcanic products around 30% porosity^18, 19, 25^, below which rocks are considered impervious, while the percolation threshold for porous media has been mathematically modelled to 59.27% in 2D^26^ and to 31.16% porosity in 3D^27^ (with circular, and spherical pores, respectively). However, other efforts have demonstrated that fluid flow is promoted at lower porosities by fractures^19, 28–33^, and hence it may not be appropriate to incorporate a percolation threshold when describing the relationship of porosity and permeability. Rather, it may be necessary to use several Kozeny coefficients^16^ due to the presence of vesicles (bubbles) and fractures^15, 18, 22, 34^, and their evolution through multiple processes [including: vesiculation^35^, shearing^30, 36, 37^, fracturing^4, 38, 39^, cooling^40^] that force pore coalescence. To describe this complexity Farquharson *et al*.^17^ proposed that the power law describing the permeability-porosity relationship can be decomposed into two regimes; a dense regime (<14 vol. % pores) for which the permeability is controlled by the connectivity of micro-fractures in the rock and a porous regime (>14 vol. % pores) for which vesicles control fluid flow. Such change points have been noted in other lithologies^41^, and yet these resolutions still fail to capture the fluid flow in natural volcanic environments (and associated hydrothermal/geothermal systems), which is channelled through structurally complex pathways, containing highly variable, heterogeneous, and anisotropic porous networks, overprinted by complex fracture networks that enhance connectivity across all scales^42–45^. The effect of fractures on the overall permeability of a rock depends on the fracture’s characteristics^46^ (e.g., size, roughness), the fracture system’s geometry^1, 47^ (i.e., direction of the fault with respect to the fluid flow), whether the fracture system is dilatant versus compactional^48–50^, and whether the fracture has in-filled fragmental material^32, 51, 52^. The presence of fractures can induce permeability anisotropy by opening localised pathways for fluid flow^1, 28, 46–48, 53^, for example, as observed along the shear margins of ascending magma^29^. Even prior to macroscopic failure, the nucleation, propagation and coalescence of micro-fractures as material is loaded (and strained) increases the permeability, and permeability anisotropy of rocks^54, 55^. The development of permeability anisotropy through damage accumulation^56–58^ can alter intrinsic properties of geothermal, hydrothermal and magmatic reservoirs, including the mode of heat transfer/fluid flow^59^. To understand the impact of macro-fractures, Lucia^60^, modelled the permeability of a system made of impermeable cubic samples separated by fractures with variable widths and determined that fracture spacing has a significant impact on the permeability of the system. In light of the importance of fractures on the development of permeable fluid flow, we hereby present the results of a series of experiments tackling the effect of fractures on permeability in rocks with variable initial porous structures (and starting permeabilities) and model the extensive dataset by adapting this cubic method^60^ to account for fluid flow through fractured rocks.

## Material and Methods

In order to assess the influence of fractures on permeability of rocks with a range of initial permeable porous networks (consisting of micro-fractures and vesicles), we selected a variety of extrusive volcanic rocks from six volcanoes (Ceboruco, Mexico; Volcán de Colima, Mexico; Krafla, Iceland; Mount St. Helens, USA; Pacaya, Guatemala; Santiaguito, Guatemala), and tested their permeability, both intact and fractured, as a function of effective pressure (calculated as the difference between the confining pressure and the average pore pressure).

70 cylindrical rock discs, 26 mm diameter and 13 mm thick were cored and prepared from the samples collected. The porosity of each disc was then calculated using quantification of the samples’ volume (based on their dimensions) and determination of the samples skeletal volume using an AccuPyc 1340 helium pycnometer from Micromeritics with a 35 cm^3^ cell (providing sample volumes with an accuracy of ±0.1%). Permeability of the variously porous (1.2–41.7 vol. %) samples was then measured under ambient pressure, using a handheld TinyPerm II mini-permeameter^61, 62^ from New England Research Inc., which utilises the pulse decay method by imposing air flow (746.13 ml) through an aperture of 8 mm (in contact with the sample). This method provides rock permeability determination with an accuracy >0.2 log units of permeability at low porosities, to 0.5–1 log units at higher porosities (verified by our dataset which includes 6–10 repeats of each measurement, see Supplementary Information). Then, for a subset of 7 samples (with porosities spanning 1.2 to 30.0 vol. %), the permeability was measured as a function of confining pressure (5–30 MPa, at 5 MPa increments) using the steady-state flow method in a hydrostatic pressure cell developed by Sanchez Technologies. Here, confining pressure was applied by silicon oil, and water flow was induced by applying a pore pressure differential (*∆P*) of 0.5 MPa (inflow of 1.5 MPa and an outflow of 1 MPa) across the sample (i.e., at an average pore pressure of 1.25 Mpa), and the flow rate (*Q*) was measured and used to compute the permeability (*k*) using Darcy’s law:

1 $$k=\frac{Q\mu L}{A{\rm{\Delta }}P}$$

where *μ* is the water viscosity, *L* is the sample thickness and *A* is the sample cross-sectional area^5, 6^. A further six unconfined measurements were made in the hydrostatic cell for direct comparison with the ambient pressure measurements of the TinyPerm (see Supplementary Figure 2). In these measurements, a *∆P* of 0.015 MPa (inflow 0.17 MPa and outflow at atmospheric pressure of 0.155) was used, and the samples were double-jacketed to prevent fluid loss (as the inflow exceeded the confining pressure). All specimens (70 measured at ambient pressure and 7 measured under confined conditions) were then axially and perpendicularly wrapped in electrical tape before being fractured using the Brazilian tensile testing method^63^ at a displacement rate of 0.25 µm/s in an Instron 5969 uniaxial press. This technique generally induces one well-defined axial, tensile fracture through a diametrically-compressed cylinder^64^. [Note that the tape was used to prevent dislocation or shearing of the two main fragments generated by tensile testing and only samples with well-defined macro-fractures were employed in permeability analysis]. Following this, the permeability of all 70 fractured samples was measured with the TinyPerm and for the aforementioned 7 samples (initially selected for permeability measurements in the hydrostatic cell) the permeability was again measured as a function of confining pressure in the hydrostatic cell.

The relative permeability change induced by the presence of a fracture was further modelled using the theoretical formulation developed for a fractured body by Lucia^60^ and modified herein for the effect of a variably permeable host material. Finally, thin sections of the rocks were prepared using a fluorescent dyed epoxy for microstructural analysis using a UV light source in reflected mode in a DM2500P Leica microscope.

## Results

### Permeability at ambient pressure

**Figure 1:**
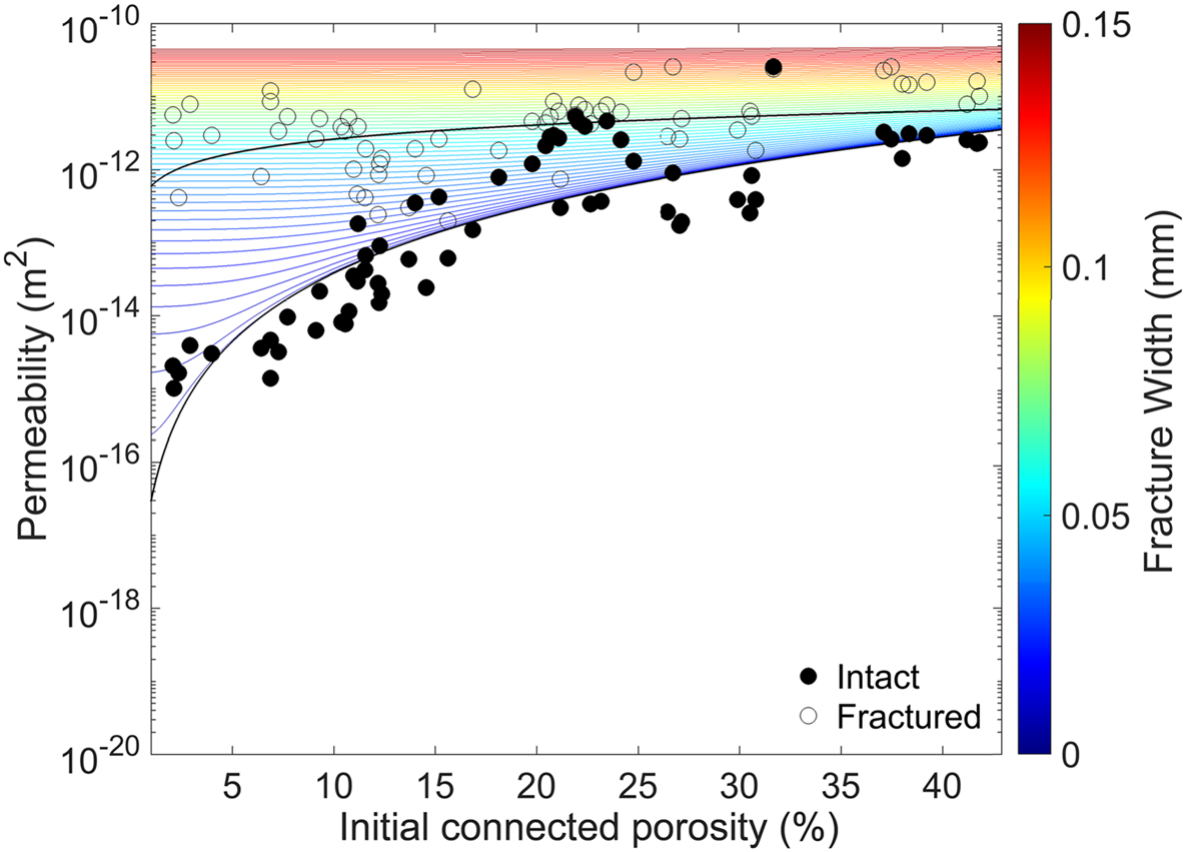
The permeability of intact and fractured rocks. Permeability-porosity relationships (black lines) for both intact (solid circles) and fractured (open circles) samples at ambient pressure. Coloured lines represent the modelled permeability of fractured rocks as a function of fracture width and rock porosity, derived from eq. 6 (See Fractured rock permeability analysis section). The convergence of the permeability values for intact and fractured samples at high porosities indicates that the effect of a fracture on permeability lessens with porosity increase, where the fluid flow is dominated by increasingly high pore interconnectivity. The data and model suggests that the fractures experimentally generated are ca. 0.06–0.07 mm wide.

We observe that permeability varies as a function of porosity, increasing by approximately four orders of magnitude (at ambient pressure) for intact samples across the range of porosities tested (1.2–41.7%; Fig. 1). This non-linear relationship between permeability (*κ*) and porosity (*Φ*), can be described by:

2 $$\kappa =3\times {10}^{-17}{\Phi }^{3.11}$$

which constrains the dataset with a coefficient of determination (R^2^) of 0.75. This relationship agrees well with that described in previous studies^18, 19^, and suggests that it is not necessary to fit this dataset with two regressions.

Using Brazilian tensile tests, we imparted a macro-fracture which resulted in a net increase in permeability for all porosities tested (Fig. 1). Across the range measured, the variability in permeability as a function of porosity (four orders of magnitude prior to fracturing) decreased to less than 2 after imparting a macro-fracture (Fig. 1). The permeability of the fracture-bearing rocks (*κ*_*fr*_) as a function of initial porosity is described by:

3 $${\kappa }_{fr}=6\times {10}^{-13}{\Phi }^{0.64}$$

Ultimately, the presence of a fracture modifies the relationship between permeability and porosity, with the permeability of fractured porous samples falling across a much narrower range than the permeability of the intact samples (i.e. much less sensitive to the initial rock porosity; Fig. 1). In detail, we note a relative increase in permeability of up to four orders of magnitude by imparting a fracture, as noted in previous work^33, 63^. This increase is most pronounced for samples with low initial porosity (≤11 vol. %). Contrastingly, the permeability of the more porous rocks (≥18 vol. %) increases only slightly due to the presence of a macro-fracture, while intermediate porosity samples (11–18%) show variable behaviour.

### Permeability at variable effective pressures

**Figure 2:**
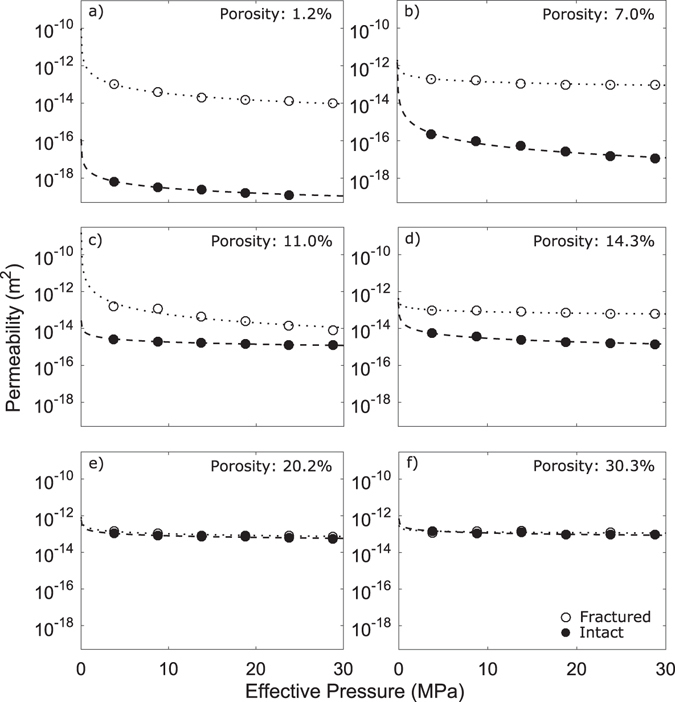
Rock permeability as a function of effective pressure. The data show the relationship between permeability and effective pressure for 6 of the 7 samples (intact and fractured) with (**a**) 1.2% porosity, (**b**) 7.0% porosity, (**c**) 11.0% porosity, (**d**) 14.3% porosity, (**e**) 20.2% porosity, and (**f**) 30.3% porosity. The impact of fracturing on a system’s permeability is much more pronounced at lower porosities than at higher porosities. Results show that the effect of a fracture on permeability is dampened with an increase in effective pressure (beyond ca. 5–10 MPa), as shown by extrapolation of the best fit (dotted and dashed curves) of the permeability dataset conducted with the pressure vessel (circles). The last sample tested (porosity very close to the sample in (**e**)) is shown in Supplementary Figure 1.

**Figure 3:**
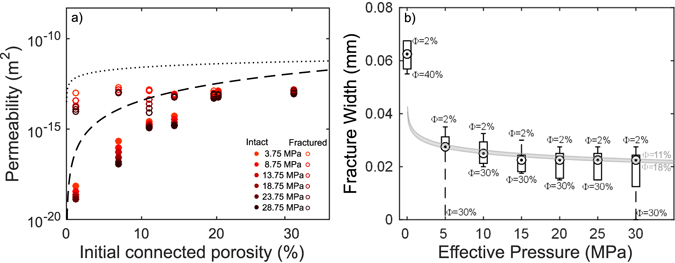
Permeability – porosity – effective pressure relationship for intact and fractured rocks. (**a**) Distribution of permeability and connected porosity data compiled as a function of effective pressure (darker colours represent higher pressures). The dashed and dotted curves display the best fits obtained for the intact and fractured samples, respectively, at ambient pressure (from Fig. 1). The measurements conducted at pressure trend towards those made at ambient pressures suggesting fracture closure even under modest confinement. (**b**) Boxplot showing the modelled fracture widths generated in samples with different porosities (Φ) and calculated evolution at different effective pressures. The grey zone displays the fracture width – effective pressure region for the porosity range 11–18 vol. %, using a least squares regression. The circles show the median of the fracture width distribution obtained by finding the closest value of the best fit, at each pressure step, to the calculated fracture width for our range of porosity.

For the subset of samples measured in the hydrostatic cell, the permeability of intact and fractured rocks decreases non-linearly with increasing effective pressure (Fig. 2; see also Supplementary Fig. 1). When plotting the data from the hydrostatic cell in porosity-permeability space, we observe similar trends to that measured at atmospheric pressure (Figs 1, 3a, Supplementary Fig. 3). We demonstrate a generally good agreement between measurements made using the handheld TinyPerm device and the hydrostatic cell by conducting a targeted set of measurements at ambient pressure in the hydrostatic cell (see Supplementary Fig. 2).

The influence of a macro-fracture on the permeability of the rocks tested here is similar at higher effective pressures as it is at atmospheric pressure, with the permeability increase that results from fracturing being more significant in the initially denser rocks (Fig. 3a). We further see that the influence of effective pressure on permeability is most pronounced in the densest rocks (≤11% porosity), while more porous rocks (≥18%) are less susceptible to changes in pressure (Figs 2, 3a); this supports previous studies, which examined the influence of pore closure under confining pressure on a range of rock types, suggesting the process is dominated by the closure of micro-fractures^4, 65–70^.

### Microstructures in intact samples

**Figure 4:**
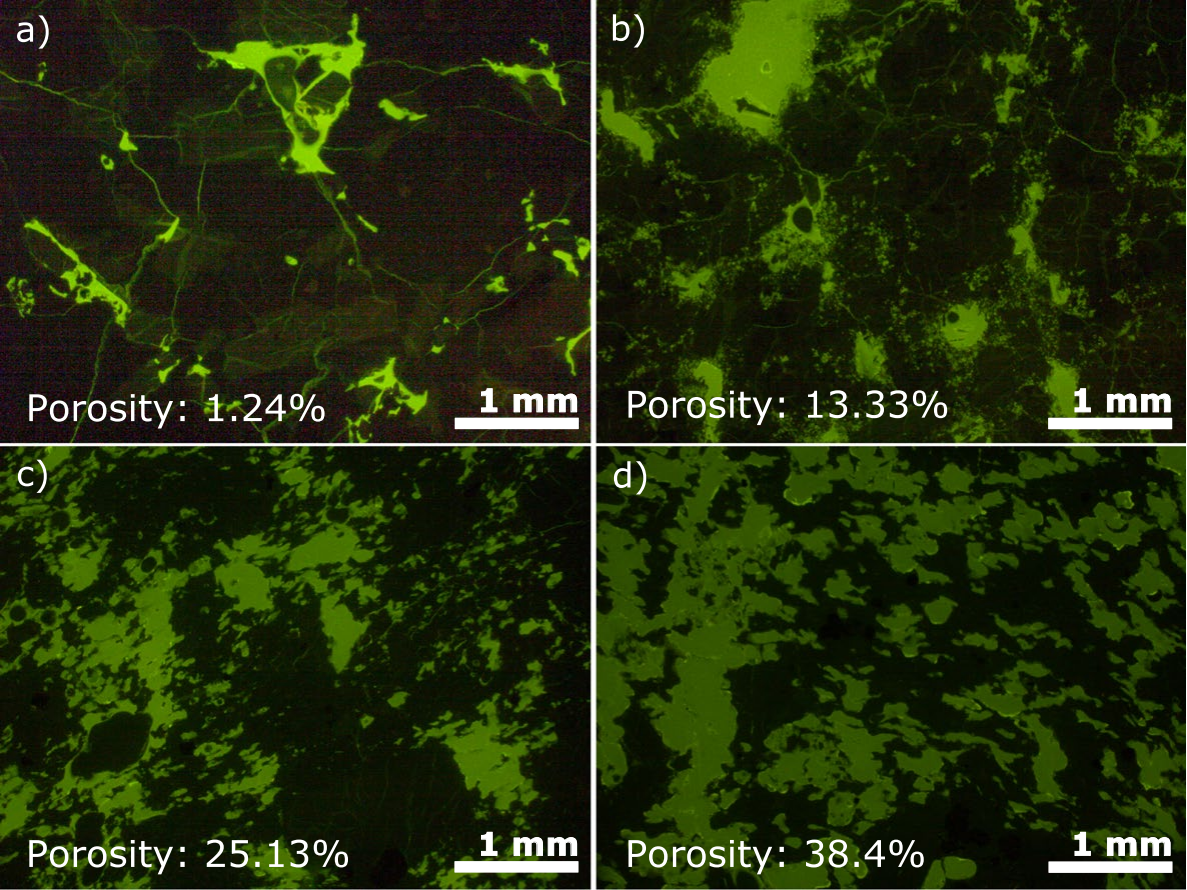
Microstructures of the permeable porous networks. Photomicrographs of 4 samples with varying connected porosities impregnated with green dyed, fluorescent epoxy, examined under UV light. (**a**) The connectivity of the densest rock, an andesite from Ceboruco (CBD_0; 1.2% porosity) is primarily controlled by micro-fractures; (**b**) The porous network of a Colima andesite with an intermediate porosity (COL_P2; 13.3%) showing a higher number of vesicles, connected to each other by micro-fractures; The connectivity of the more porous rocks from Ceboruco, (**c**) an andesite with 25.1% porosity (CBD_6); (**d**) an andesite with 38.4% porosity (CBD_10) is observed to be primarily controlled by vesicle coalescence.

Microstructural analysis was conducted on thin sections impregnated with fluorescent green-dyed epoxy (highlighting the porous network of the rocks) to assess the reasons for the relative impact of a fracture on volcanic rocks at low and high porosities (Fig. 4). The rocks tested here were chosen for their chemical and mineralogical distinctions so as to widen the applicability of the findings of the influence of the porous network on permeability accross a range of volcanic rocks and environments. The porous networks of the densest rocks (Fig. 4a,b) are dominated by an intricately connected network of micro-fractures, linking the vesicles present in the rock^71^. Close examination of the photomicrographs show no overall preferential alignment (i.e., anisotropy) of the microfractures, but do highlight preferred fracture developments along planes of weakness in phenocrysts. In contrast, the porous networks of the more porous rocks (Fig. 4c,d) appear dominated by the connectivity of vesicles of different sizes and shapes. These porous rocks exhibit few microfractures, and those which are present are primarily developed in phenocrysts (Fig. 4c,d). Such a contrasting architecture of the porous networks in dense and porous volcanic rocks has been observed in other studies^24, 33, 72^ and may be at the origin of the non-linearity in permeability-porosity relationships discussed in previous studies^17, 24, 72^ and in the relative effect of a fracture on the permeability of rocks as observed here. As such, we seek to test the applicability of fracture permeability modelling to describe the permeability relationships constrained in our experiments.

### Fractured rock permeability analysis

The permeability of fractures as a function of width can be modelled using the early work of Lucia^60^, in which the geometrical proportion of a fracture set arrangement is applied to a cubic body. The relationship is based on the principal of a pressure differential (*∆P*) across a fracture with given length (*L*) and width (*w*), according to:

4 $$\Delta P=\frac{12\mu \nu L}{{w}^{2}}$$

where *μ* and *v* are the viscosity and velocity of the fluid flowing through the fracture, respectively. Lucia^60^ later modified the equation to obtain a system permeability (*κ*_*s*_) formulation, which includes the area of the fracture as well as the surrounding rock:

5 $${\kappa }_{s}=\frac{1}{12}\frac{{A}_{f}}{{A}_{s}}{w}^{2}$$

where *A*_*f*_ and *A*_*s*_ are the cross sectional areas of the fracture and the sample, respectively. Considering the host rock permeability (*κ*_*Φ*_), our cylindrical sample geometry and the near rectangular fracture geometry (produced in this study through Brazilian tests), Equation 5 can be further modified to:

6 $${\kappa }_{s}={\kappa }_{\Phi }+\frac{1}{6}\frac{{w}^{3}}{\pi r}$$

in which *κ*_*Φ*_ is the permeability of intact samples (each at a given porosity) and *r* is the aperture radius of the permeameter (i.e., 4 mm for the TinyPerm and 13 mm for the hydrostatic cell).

Using this relationship, we model the macro-fracture width (i.e., the coloured curves in Fig. 1) for rocks with different initial porosities and permeabilities. The permeability measurements on fractured samples coincide with the modelled permeability for rocks hosting a fracture of some 0.06–0.07 mm wide. We apply this analysis to the permeability obtained at each effective pressure (Fig. 3a, Supplementary Fig. 3), to constrain the evolution of fracture width as a function of effective pressure. The boxplot (Fig. 3b) shows the modelled fracture widths for our range of porosities with increasing pressure. All boxes have been defined by finding the closest modelled fracture width to each permeability measurement at each effective pressure (see Fig. 1 and Supplementary Fig. 3). The analysis suggests that the fracture closes non-linearly with effective pressure^73^, corresponding to the measured non-linear decrease in permeability, with most of the fracture closure occurring within the first 5 MPa of confinement for all samples, irrespective of initial porosity (Fig. 3b).

In light of this constraint, and given the knowledge of the bulk fracture density (volume of macro-fracture/volume of host rock), we rewrite the above permeability equations to provide a general formulation for the permeability of a fractured system (*κ*_s_) as a function of the permeability of the intact system﻿ (κ_Φ_), bulk fracture density (*ρ*_*f*_), average fracture length ($\bar{l}$) and width ($\bar{w}$) ﻿over an area of interest (*A*_*i*_):

7 $${\kappa }_{s}={\kappa }_{\Phi }+\frac{{\rho }_{f}\bar{l}{\bar{w}}^{3}}{{A}_{i}}$$

In this formulation, the left-hand term expresses the permeability evolution of the system as a function of effective pressure and porosity, whilst the right-hand term constrains the impact of fractures on the overall permeability of the system.

We can further expand this formulation to include the empirical description of the effect of effective pressure on the permeability of the intact rock (Eq. 8) as well as on the fracture width (Eq. 9; see equations S2–7 in Supplementary Information)

8 $${\kappa }_{\Phi }=(2.93\times 10{}^{-12}{{P}_{eff}}^{-1.07}){\Phi }^{(1.64{{P}_{eff}}^{0.06})}$$

And

9 $$w=(2.33\times {10}^{-22}{{P}_{eff}}^{2}-2.67\times {10}^{15}{P}_{eff}+3.39\times {10}^{-7}){\Phi }^{(5\times {10}^{-4}{{P}_{eff}}^{-0.174})}$$

where ${P}_{eff}$ is the effective pressure in Pascals and each coefficient has different pressure dependent unit described in Supplementary Information. Thus we can rewrite Equation 7 to:

10 $$\begin{array}{c}{\kappa }_{s}=(2.93\times 10{}^{-12}{P}_{eff}^{-1.07}){\Phi }^{(1.64{P}_{eff}^{0.06})}\\ \quad \quad +\,\frac{{\rho }_{f}\bar{l}{[(2.33\times {10}^{-22}{P}_{eff}^{2}-2.67\times {10}^{15}{P}_{eff}+3.39\times {10}^{-7}){\Phi }^{(5\times {10}^{-4}{P}_{eff}^{-0.174})}]}^{3}}{{A}_{i}}\end{array}$$

providing us with an empirical description of rock permeability as a function of effective pressure, porosity, fracture density and geometry to be tested in various applications.

## Discussion

Understanding the permeability of volcanic rocks, and especially fractured volcanic rocks, is crucial to our models of fluid flow in shallow volcanic and hydrothermal systems^2, 74^. Here, a combination of extensive permeability testing and fluid flow modelling is used to demonstrate the ability to simulate the permeability of intact and fractured rocks and of fracture closure with confinement. In our fitting of the permeability-porosity relationship, we employed a single power law (as demonstrated by previous studies^15, 18, 19, 22, 34^) as the regression is sufficient to fit the non-linear dataset accurately, without the need to invoke a change point. From microstructural examination (Fig. 4), we find that the connectivity of the porous network evolves due to the interplay of micro-cracks and few vesicles at low porosity, to enhanced pore interconnection at 11–18% porosity (an observation which may share similarities with previously invoked change points^17^) and finally more complete coalescence at porosities ≥18%. We emphasise that the porosity-permeability relationship of volcanic rocks results from a succession of processes undergone by the magma and the rock (i.e., vesiculation and pore collapse, fragmentation, sintering, shearing, cooling, contraction, etc) and as a result the porosity-permeability relationship does not describe a single generation mechanism, but rather reflects a combination of the above, which may have differing importance at different porosities. As permeability measurements accrue and widen the scatter at all porosities, evidence suggests that a simple power law, with acknowledgement of the scatter, remains an effective means to estimate the permeability of volcanic systems with wide ranging porous structures.

Across the range of porosities tested, the presence of a macro-fracture increases the permeability of volcanic rocks, although to different degrees, depending on the porosity of the rock. The impact of fractures on the resultant system permeability is greatest for low porosity rocks, where permeability can increase by up to four orders of magnitude, which can be ascribed to a decrease in the tortuosity of the dominant fluid pathway by addition of a macro-fracture^63^. This increase in permeability as a result of fracturing has previously been noted^33, 52, 75^. Here, we show that the initial porosity of the samples has little influence on the resultant system permeability once a fracture is introduced. Matthäi and Belayneh^76^ classified the influence of a fracture on a rock permeability as either 1) fracture carries all the fluid flow; 2) fracture carries as much fluid flow as the host rock; or 3) fracture has a negligible impact on the permeability. Based on the findings presented here, we relate this classification to the relative magnitudes of permeability changes imparted by a fracture on rocks with different porosities: Regime 1 relates to dense rocks with ≤11% porosity; regime 2 to rocks with ~11–18% pores and regime 3 to the most porous rocks (≥18%), in which the presence of a macro-fracture imparts little change on the permeability of the system (Fig. 3). Interestingly, we find that the porosity thresholds for regime changes remain unaffected by changes in effective pressure, although the magnitude of permeability increase by inducing a fracture (i.e. the fracture width) is itself pressure dependent.

We provide an experimentally based, permeability model to describe the permeability of macro-fractured volcanic rocks with a range of existing permeable porous structures, which, using appropriate upscaling techniques^33, 77, 78^, may be adapted to a range of geological systems^60^. Utilisation of the simple formulation provided may help constrain or reassess a variety of processes for which an understanding of fluid flow pathways developed via multiple processes is crucial. For example, the percolation threshold of explosive volcanic products^18, 19, 25^ may be modified significantly by fracturing. Previous works have demonstrated that outgassing in volcanic materials occurs through a network of fractures that localise and enhance fluid flow^19, 28–33^, and gas monitoring at active volcanoes supports heterogeneous degassing models controlled by fractures in often low-permeability host rocks^74^. Further, at the volcano-hydrothermal system of Soufrière Hills volcano (Montserrat), Edmonds *et al*.^74^ surmise that cyclicity/fluctuations in gas emissions result from fractures undergoing episodic closure or sealing, leading to permeability changes in regions with high permeability anisotropy near conduit margins^28, 29, 79^. Our findings concur with these outgassing observations, as pore pressure (hence effective pressure) regulates the permeability of intact and fractured rocks. In this scenario, efficient outgassing may promote the lowering of pore pressure (i.e., effective pressure increase), fostering the ability for fractures to shut and subsequently heal^80^. It must be noted that this sealing will be dependent upon any fracture infill, which may either form a rigid network serving to maintain the permeable pathway, or may be subject to compaction or sintering, influencing the evolution of permeability^32, 52^. Sealing may inhibit further fluid flow and promote creation of momentarily impermeable, dense magma plugs^30, 74, 81^, which may then allow pore pressure build-up (i.e., effective pressure decrease), which if sufficient, may open (or reactivate) fractures or trigger fragmentation^82^. Thus, we advise testing of the formulation constrained here in anticipation that it may increase constraints on fluid migration and storage in volcanic, hydrothermal and geothermal systems.

## Conclusions

We present a large permeability dataset, targeted to investigate the effects of porosity, fractures and effective pressure on the permeability of variably porous volcanic rocks. We observe non-linear relationships between porosity and permeability of both intact and fractured rocks as well as between the width of a fracture (and permeability of a fractured rock) and effective pressure. We propose a general formulation to constrain the permeability of intact and fractured rocks as a function of pressure, porosity and fracture density. This study aims to incorporate heterogeneities, such as fractures, in our modelling of the permeability evolution of dynamic and heterogeneous volcanic environments.

## Electronic supplementary material

Supplementary Information

K_Peff_data

w_Peff_data
