# Supplementary material for: The permeability of fractured rocks in pressurised volcanic and geothermal systems
Source: Sci Rep. 2017 Jul 21;7:6173. doi: 10.1038/s41598-017-05460-4 (PMC5522408; doi:10.1038/s41598-017-05460-4)
Supplement: Supplementary file 1 — Supplementary Information [file 41598_2017_5460_MOESM1_ESM.docx]

The permeability of fractured rocks in pressurised volcanic and geothermal systems

**A Lamur*, J.E. Kendrick, G.H. Eggertsson, R.J. Wall, J.D. Ashworth, Y. Lavallée**

# Supplementary information

| **Volcano** | **Bulk chemistry** | **Porosity range (%)** | **Age (yrs)** | **Sample name** |
| --- | --- | --- | --- | --- |
| Ceboruco, Mexico | Andesite | 2-4 | 100-1000 | CBD_0 |
|  |  | 12-15 | 100-1000 | CBD_9 |
|  |  | 14 | >1000 | CBLF |
|  |  | 19-22 | 100-1000 | CBD_19 |
|  |  | 22-26 | 100-1000 | CBD_6 |
|  |  | 29-32 | 100-1000 | CBD_7 |
|  |  | 37-41 | 100-1000 | CBD_10A |
| Colima, Mexico | Andesite | 11-14  17-23 | <100  <100 | COL_P2  COL_P21 |
| Krafla, Iceland | Basalt | 11 | <100 | KRA_BAS |
| Mount St Helens, USA | Dacite | 9-12 | <100 | MSH_20 |
| Pacaya, Guatemala | Basalt | 6-9 | >1000 | PAC_L_SCARP |
|  |  | 12-14 | <100 | PAC_2010 |
|  |  | 18-23 | <100 | PAC_BA |
|  |  | 24-30 | <100 | PAC_J13 |
| Santiaguito, Guatemala | Andesite | 19-21 | <100 | SGLF4 |
|  |  | 35-41 | <100 | SGPF2 |

**Supplementary Table 1.** Origin, bulk chemistry, porosity range and age of the samples used in this study. Porosity is given as a range when several samples (2 to 8) were cored and measured from the same rock.

**Supplementary Figure 1**


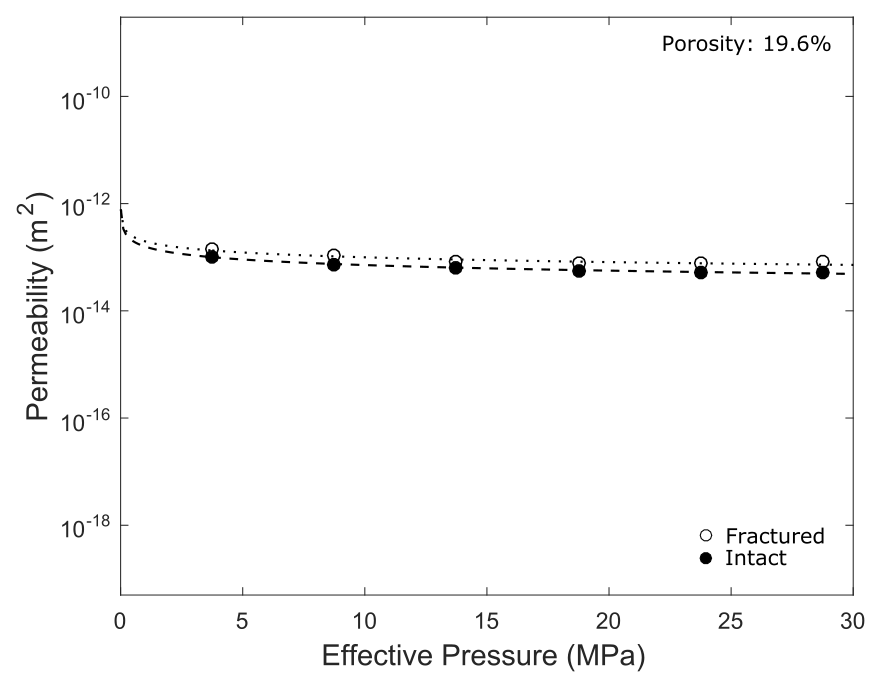


**Suppl. Fig. 1.** Rock permeability as a function of effective pressure. The data show the relationship between permeability and effective pressure for the 19.6% porosity sample tested in the hydrostatic cell. The results for the intact and fractured sample show similar permeability-effective pressure relationships to those shown in Fig. 2e.

**Supplementary Figure 2**


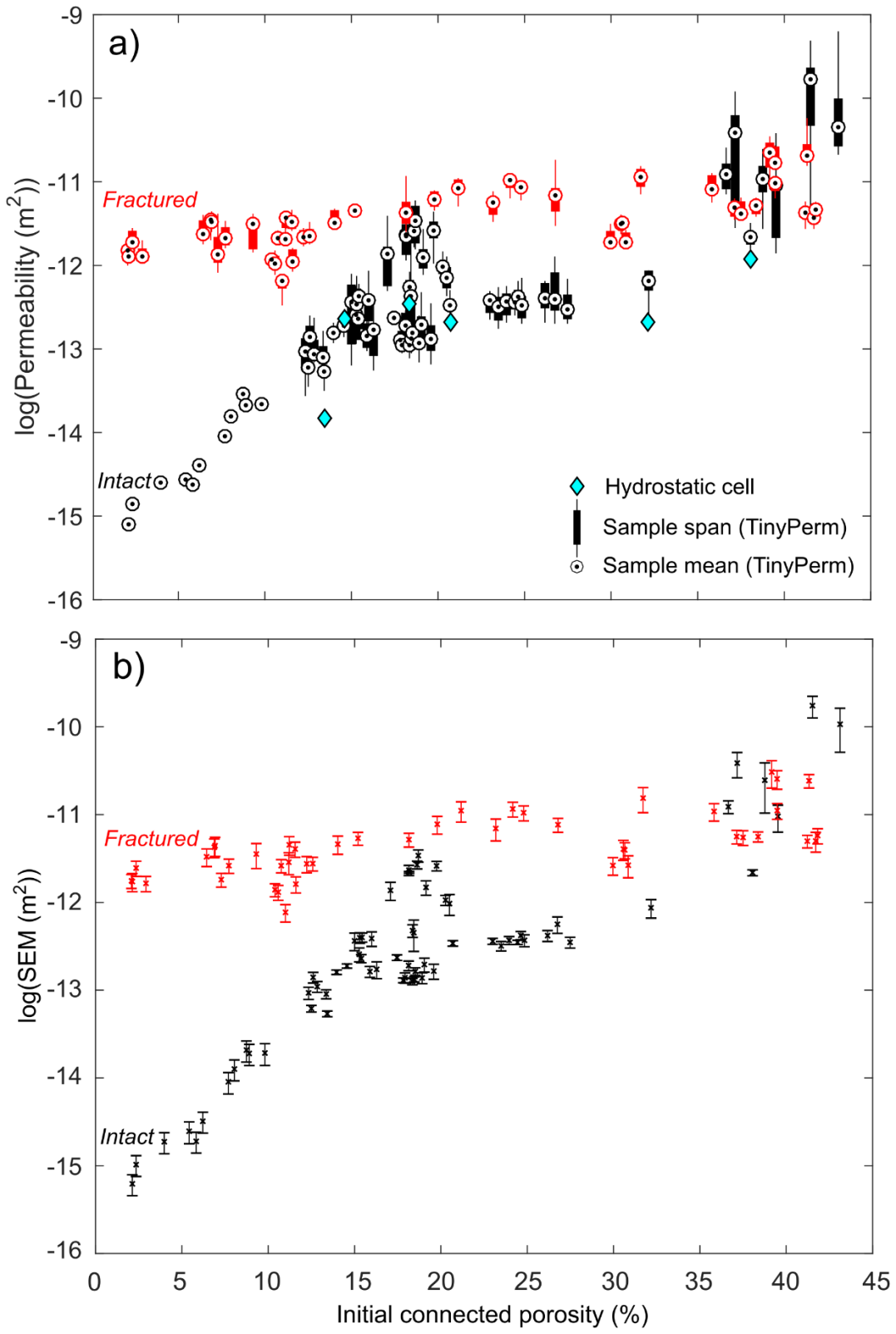


**Suppl. Fig. 2.** a) Boxplot showing the span (boxes) and the mean permeability (dotted circles) of 111 samples measured with the TinyPerm. Note that black symbols show the reproducibility of the method on intact samples, while red symbols show measurement variability on fractured samples. Each box represents the span obtained from up to 10 repetitions on fractured and intact samples. Samples with porosity below 10% were measured 6 times due to their high reproducibility (span smaller than the mean symbol) and the time required for 1 measurement. The boxes indicate results within 1 standard deviation of the mean and lines show the 2^nd^ to the 98^th^ percentile of the distribution of measurements. b) Shows the Standard Error from the Mean (SEM) for each of the measurements.


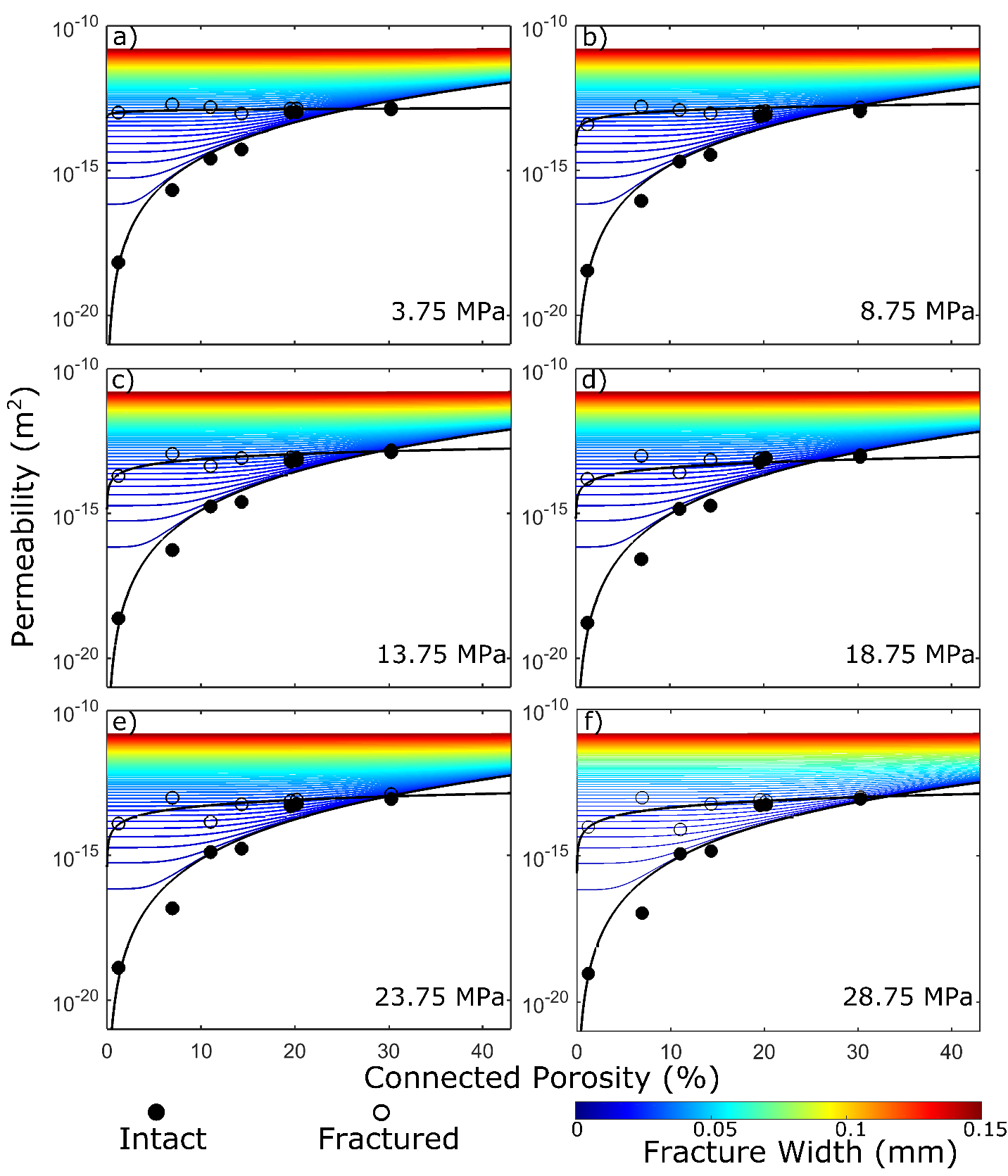
**Supplementary Figure 3**

**Suppl. Fig. 3.** Permeability-porosity relationships (black lines) derived from fitting a least-squares function to the intact (solid circles) and fractured (open circles) samples measured in the hydrostatic pressure cell at different effective pressure steps (shown in panels a-f). Coloured lines represent the calculated fracture widths as a function of starting porosity, derived from eq. 6. (See Analysis section). This demonstrates that increasing effective pressure reduces the fracture width, and hence permeability, across all starting porosities tested, although the influence is more pronounced at lower porosities.

# Empirical solution for Equation 7

All data used to determine an empirical solution can be found in the attached files K_Peff_data.xls and w_Peff_data.xls

$\kappa_{s}=\kappa_{\Phi}+\frac{\rho_{f}\overline{l}\overline{w}^{3}}{A_{i}}$ (S1)

Equation 7 in the manuscript, that expresses the permeability evolution of a fractured system ${(\kappa}_{s})$ with effective pressure solvable, in terms of the intact rock permeability ${(\kappa}_{\Phi})$, fracture density ${(\rho}_{f})$, average fracture length $( \overline{l})$ and width $( \overline{w} )$ over an area of interest ${(A}_{i})$. Hence, equation 7 is solvable when the evolution of $\kappa_{\Phi}$ is known.

Our dataset however can provide an empirical solution to estimate permeability evolution of laboratory samples as a function of effective pressure and porosity from direct measurements. Furthermore, using the fracture width modelling, our results can provide an approximation of fracture closure with depth, necessary to compute the fractured system permeability.

Despite physical modelling, this empirical solution relies on measured data and can aid with modelling the permeability evolution of porous media with depth as well as the impact of fractures on the same systems:

1. Least-squares functions for the permeability evolution of intact porous media and fracture closure as a function of effective pressure:

| **Peff (Pa)** | $\boldsymbol{\kappa}_{\boldsymbol{\Phi}}$ | $w$ |
| --- | --- | --- |
| 150000 | $9.14\times{10}^{-18} \Phi^{3.16}$ | $-4.17\times{10}^{-7}\Phi+7.03\times{10}^{-5}$ |
| 3750000 | $1.87\times{10}^{-18} \Phi^{4.03}$ | $-2.48\times{10}^{-7}\Phi+3.33\times{10}^{-5}$ |
| 8750000 | $9.41\times{10}^{-18} \Phi^{4.15}$ | $-4.10\times{10}^{-7}\Phi+3.31\times{10}^{-5}$ |
| 13750000 | $6.30\times{10}^{-20} \Phi^{4.20}$ | $-4.09\times{10}^{-7}\Phi+2.96\times{10}^{-5}$ |
| 18750000 | $4.73\times{10}^{-20} \Phi^{4.21}$ | $-4.29\times{10}^{-7}\Phi+2.90\times{10}^{-5}$ |
| 23750000 | $3.77\times{10}^{-20} \Phi^{4.17}$ | $-4.44\times{10}^{-7}\Phi+2.87\times{10}^{-5}$ |
| 28750000 | $3.20\times{10}^{-20} \Phi^{4.29}$ | $-5.15\times{10}^{-7}\Phi+2.85\times{10}^{-5}$ |

**Supplementary Table 2.** Permeability evolution of intact porous media${(\kappa}_{\Phi}$) and fracture width $(w)$ with increasing effective pressure.

1. Determining empirical functions for the evolution of the different coefficients for intact rock permeability with effective pressure. (Data in K_Peff_data.xls: Tab Coefficients eq. det.)

The equations determining $\kappa_{\Phi}$ are all expressed as power laws ($a$*x^b^*), with $a$ and $b$ respectively decreasing and increasing with effective pressure following these equations:

$a=2.93\times{10}^{-12}{P_{eff}}^{-1.07}$ (S2)

$b=1.64{P_{eff}}^{0.06}$ (S3)

In these equations S2-3, the coefficients have different units: 2.93x10^-12^ m^2^.Pa^1.07^ and 1.64 Pa^0.06^. Hence, the evolution of $\kappa_{\Phi}$ as function of porosity and effective pressure can be expressed by the empirical law:

$\kappa_{\Phi}=\left( 2.93\times{10}^{-12}{P_{eff}}^{-1.07} \right)\Phi^{\left( 1.64{P_{eff}}^{0.06} \right)}$ (S4)

Where $P_{eff}$ is expressed in Pascals.

1. Determining empirical functions for the evolution of the different coefficients for fracture closure with effective pressure. (Data in w_Peff_data.xls: Tab Coefficients eq. det.)

Similarly, we can see that for each effective pressure tested, $w$ follows a linear law (*c x + d*) as a function of porosity, where *c* expresses the fracture closure and *d* the fracture width for theoretical samples with 0 % porosity. The evolution of *c* across our range of porosities follows a second order polynomial function with increasing effective pressure, suggesting that the amount of closure with increasing effective pressure is more important at higher porosities. On the other, d shows a power law dependence with increasing effective pressure.

$c=2.33\times{10}^{-22}{P_{eff}}^{2}-2.67\times{10}^{15}P_{eff}+3.39\times{10}^{-7}$ (S5)

$d=5\times{10}^{-4}{P_{eff}}^{-0.174}$ (S6)

In these equations S5-6, the coefficients have different units: 2.33x10^-22^ m.Pa^-2^, 2.67x10^-15^ m.Pa^-1^, 2.23x10^-7^ m, 5x10^-4^ m.Pa^0.174^. The evolution of fracture width with increasing effective pressure can then be expressed as a function of porosity and effective pressure:

$w=\left( 2.33\times{10}^{-22}{P_{eff}}^{2}-2.67\times{10}^{15}P_{eff}+3.39\times{10}^{-7} \right)\Phi^{\left( 5\times{10}^{-4}{P_{eff}}^{-0.174} \right)}$ (S7)

Considering a constant fracture length (in our case 0.0026 m), or a fracture aspect ratio (w/l), we can then empirically solve equation 7:

$\kappa_{s}=\left( 2.93\times{10}^{-12}{P_{eff}}^{-1.07} \right)\Phi^{\left( 1.64{P_{eff}}^{0.06} \right)}+\frac{\rho_{f}\times l\times\left[ \left( 2.33\times{10}^{-22}{P_{eff}}^{2}-2.67\times{10}^{15}P_{eff}+3.39\times{10}^{-7} \right)\Phi^{\left( 5\times{10}^{-4}{P_{eff}}^{-0.174} \right)} \right]^{3}}{A_{i}}$ (S8)

Where $A_{i}$ expresses the size of the pore pressure source and $\rho_{f}$ is defined by:

$\rho_{f}=\frac{\overline{l}\overline{w}}{A_{i}}$ (S9)

This empirical formulation, allows resolution of the permeability evolution of a fractured porous medium with increasing effective pressure. We draw the reader’s attention to the fact that by being empirical, this formulation, while providing a first order approximation of permeability, should be carefully tested and calibrated by changing experimental conditions.
